# Proteome-Wide Search Reveals Unexpected RNA-Binding Proteins in *Saccharomyces cerevisiae*

**DOI:** 10.1371/journal.pone.0012671

**Published:** 2010-09-10

**Authors:** Nikoleta G. Tsvetanova, Daniel M. Klass, Julia Salzman, Patrick O. Brown

**Affiliations:** 1 Department of Biochemistry, Stanford University School of Medicine, Stanford, California, United States of America; 2 Howard Hughes Medical Institute, Stanford University School of Medicine, Stanford, California, United States of America; 3 Department of Statistics, Stanford University, Stanford, California, United States of America; Texas A&M University, United States of America

## Abstract

The vast landscape of RNA-protein interactions at the heart of post-transcriptional regulation remains largely unexplored. Indeed it is likely that, even in yeast, a substantial fraction of the regulatory RNA-binding proteins (RBPs) remain to be discovered. Systematic experimental methods can play a key role in discovering these RBPs - most of the known yeast RBPs lack RNA-binding domains that might enable this activity to be predicted. We describe here a proteome-wide approach to identify RNA-protein interactions based on *in vitro* binding of RNA samples to yeast protein microarrays that represent over 80% of the yeast proteome. We used this procedure to screen for novel RBPs and RNA-protein interactions. A complementary mass spectrometry technique also identified proteins that associate with yeast mRNAs. Both the protein microarray and mass spectrometry methods successfully identify previously annotated RBPs, suggesting that other proteins identified in these assays might be novel RBPs. Of 35 putative novel RBPs identified by either or both of these methods, 12, including 75% of the eight most highly-ranked candidates, reproducibly associated with specific cellular RNAs. Surprisingly, most of the 12 newly discovered RBPs were enzymes. Functional characteristics of the RNA targets of some of the novel RBPs suggest coordinated post-transcriptional regulation of subunits of protein complexes and a possible link between mRNA trafficking and vesicle transport. Our results suggest that many more RBPs still remain to be identified and provide a set of candidates for further investigation.

## Introduction

The dynamic processes of a living cell depend on the coordinated temporal and spatial regulation of the many steps of gene expression. Combinatorial binding and control of gene transcription by specific transcription factors allows for individual regulation of each gene and concerted regulation of large sets of genes in physiological and developmental programs. While transcription is a major control point of gene expression, a gene's transcript can also be subject to regulation at the levels of RNA processing, transport, localization, translation, and degradation. The correlation between mRNA transcript abundance and protein abundance was only 0.5–0.6 in a survey of 80% of the yeast genome, suggesting significant post-transcriptional regulation [1]. Similar conclusions have been drawn from a comparison of changes in mRNA transcript abundance to changes in protein abundance in response to a shift in growth media [2].

Recent work further corroborates the existence of extensive post-transcriptional regulatory networks, with an ever-growing list of specific RNA binding proteins (RBPs) that bind distinct sets of mRNAs encoding proteins destined for similar subcellular locations or with similar biological functions [3], [4], [5], [6], [7]. Nevertheless, very little is known about the specific pathways involved in the posttranscriptional regulation of gene expression or their molecular components.

One important part of defining the system that regulates the posttranscriptional fate of mRNAs in *Saccharomyces cerevisiae* is to identify all the proteins that interact with these RNAs. Currently, over 600 proteins in *S. cerevisiae* are thought to bind RNA (Table S1) [8]. Though this list of “known” RBPs is long, comprising more than 10% of the yeast proteome, some proteins not annotated as RBPs reproducibly co-immunipurify with distinct sets of RNAs *in vivo* [5]. Most of the yeast proteins annotated as RBPs (Table S1) lack domains known to bind RNA, and some RBPs have other known functions that give no hint of their involvement in the post-transcriptional regulation of RNA. For example, the metabolic enzyme aconitase catalyses the isomerization of citrate to isocitrate, but the cytosolic version also functions as an RNA binding protein, binding to iron regulatory elements in target mRNAs to regulate their expression in response to iron availability [9]; Glyceraldehyde-3-phosphate dehydrogenase (GAPDH) binds directly to AU-rich sequences in specific RNAs in humans and in *S. cerevisiae* [5], [10]; and enolase mediates the mitochondrial import of specific tRNAs as the enolase-preMSK1 [11]. These and other examples of a regulatory RNA-binding activity in unexpected proteins highlight the need for systematic experimental methods for discovering novel RBPs [12].

We describe two methods to search for novel RNA-protein interactions. The main approach used protein microarrays containing 4,700 different yeast proteins (>80% yeast proteome) to interrogate RNA-protein interactions *in vitro* on a genome-wide scale. Protein microarrays have been previously used to identify proteins that interact with a small viral RNA hairpin [13]. A complementary method combined affinity purification with mass spectrometry to identify proteins from a whole cell lysate that co-purify with total poly(A) mRNA.

## Results and Discussion

### Protein microarrays detect specific mRNA-protein interactions with high sensitivity and specificity

Our goal was to survey the majority of the yeast proteome for specific RNA-binding activity. We purified more than 4,700 GST-tagged proteins, representing >80% of the yeast proteome [14]. 451 of these 4,700 proteins are known or predicted to associate with RNA [8]. 75 of the purified proteins were picked at random and their identity verified by Western blotting (data not shown).

There were several potential sources of variation associated with the protein purification method that we used to prepare the protein microarrays. Protein-to-protein variation in expression and purification efficiency could result in differences in the amount of protein printed per spot. In addition, since all the proteins were purified from yeast cells, we cannot exclude the possibility that interacting proteins co-purified with the tagged protein nominally present at a spot. Also, difference in protein stability and variability in efficiency and manner of immobilization on the nitrocellulose microarray surface due to charge or size could affect the amount of properly folded and oriented protein in each spot. We first tested whether, despite these potential limitations, we could use the arrays to identify known specific RNA-protein interactions.

**Figure 1:**
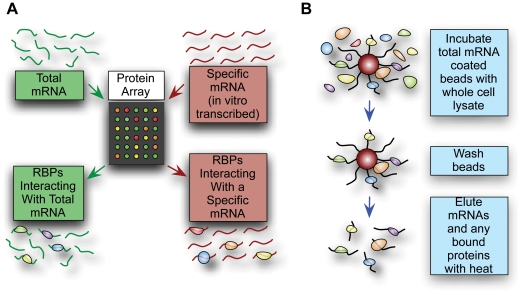
Methods for identifying novel RNA-binding proteins. **A.** A schematic representation of the protein microarray method. Specific mRNA of interest is synthesized *in vitro*, while polyA-selected mRNA is isolated from mid-log phase cells grown in YPD. The samples are labeled with Cy-dyes, pooled together and bound to a protein microarray. In subsequent analyses, proteins interacting with total mRNA or preferentially with individual mRNA are identified. **B.** Proteins associating with mRNAs immobilized on oligo-dT beads were isolated and identified using LC-MS/MS.

As a positive control, we chose the well-characterized *ASH1* mRNA, which encodes an inhibitor of mating-type switching in yeast [15], [16], [17]. During cell division, the *ASH1* mRNA is localized in a She2-dependent manner via Myo4 to daughter cell nuclei, and Khd1-binding to the *ASH1* mRNA ensures that it is not translated until properly localized ([18], [19], [20], [21] and others). A protein microarray was incubated with a mixture of fluorescently labeled *in vitro* transcribed *ASH1* mRNA and poly(A)-selected total mRNA from cells harvested at mid-log phase in YPD (Figure 1A). A total of four replicates were performed, and the Cy3 and Cy5 dyes used to label the mRNA samples were reversed between replicates.

We ranked proteins by normalized mean intensity of the fluorescent signal representing *ASH1* mRNA measured at the cognate spot in the microarray. For 42 proteins, the fluorescent signal in at least one of four replicate experiments was at least two standard deviations above the mean for all spots. Fluorescence ratios for proteins with signal below this threshold showed no rank correlation between dye swaps (Spearman correlation coefficient = 0.09). Nine of those 42 proteins had signal consistently two or more standard deviations above the mean regardless of the Cy-dye label (Spearman correlation coefficient = 0.7, p-value = 1×10^−14^, Table 1). These nine proteins were not notably deviant in charge or abundance when compared to the proteins that did not pass the threshold. Five of those nine proteins were known RNA-binding proteins (She2, Npl3, Rrp5, Khd1, Scp160), four of which have previously been reported to bind the *ASH1* transcript (Npl3, She2, Khd1, Scp160) [5], [15], [19]. The remaining four proteins (Ydl124w, Gcy1, Pcs60 and Mdh3) were enzymes not previously described to interact with nucleic acids.

**Table 1:** Proteins interacting with *ASH1* mRNA identified in replicate dye-swap protein microarray experiments.

Protein	Description	Function	Avg Cy3	Avg Rank Cy3	Avg Cy5	Avg Rank Cy5	pI	Abundance
**She2**	**RBP that interacts with She3p; restricts accumulation of certain proteins to the bud**	**mRNA binding**	**17.1**	**3**	**17.8**	**18**	**4.7**	**4,070**
YDL124W	NADPH-dependent alpha-keto amide reductase	alpha-keto ester reductase activity	11.9	5	14.6	22	6.1	4,030
Gcy1	Putative NADP(+) coupled glycerol dehydrogenase	oxidoreductase activity	8.9	8	19.6	17	8.2	NA
**Npl3**	**RBP that promotes elongation, regulates termination, and carries poly(A) mRNA from nucleus to cytoplasm; required for pre-mRNA splicing**	**mRNA binding**	**7.1**	**12**	**153.8**	**1**	**5.4**	**78,700**
**Rrp5**	**RBP; preference for tracts of U's; synthesis of both 18S and 5.8S rRNAs**	**mRNA binding**	**3.8**	**20**	**14.6**	**23**	**6.1**	**8,860**
Pcs60	AMP-binding protein; peroxisomal peripheral membrane and matrix	ligase activity	3.7	21	89.8	4	10.0	8,770
**Khd1**	**RBP that represses translation of ASH1 mRNA; regulates telomere position effect and length**	**mRNA binding**	**3.4**	**24**	**24.0**	**15**	**6.0**	**15,600**
**Scp160**	**RBP; mating response pathway; nuclear envelope and ER; interacts with translating ribosomes**	**mRNA binding**	**2.9**	**29**	**117.7**	**3**	**5.7**	**NA**
Mdh3	Peroxisomal malate dehydrogenase	dehydrogenase activity	2.9	31	60.2	8	10.0	3,300

These proteins had normalized mean signal over median background signal (for the *ASH1* Cy-dye channel) equal to or greater than two SD above the mean for each microarray experiment. Annotated RBPs are identified by bold text. *ASH1*mRNA microarray signal (Cy3 or Cy5) and rank are averaged over two replicates for each dye. Protein abundance data are taken from [1], protein isoelectric point from [8].

The greater the specificity with which a protein binds to *ASH1* in preference to other mRNAs, the higher the ratio of the fluorescent signal corresponding to the labeled *ASH1* compared to the signal representing total mRNA. She2 and Khd1 were the two proteins that showed the highest ratio of signal representing *ASH1* RNA to the signal representing total mRNA reference (Log2G/R (mean-centered) for She2 was 3.4 in *ASH1-*Cy3 experiments and 5.7 in *ASH1-*Cy5 experiments; Log2G/R (mean-centered) for Khd1 was 1.1 in *ASH1-*Cy3 experiments and 3.7 in *ASH1-*Cy5 experiments). The lower signal ratios for the remaining seven proteins suggest that they may represent RNA-binding proteins with broader specificity. Indeed, Scp160 and Npl3 appear to interact with almost the entire transcriptome, and *ASH1* mRNA was among the RNAs least enriched by affinity purification of Scp160 or Npl3 [5].

Among the five candidate *ASH1*-interacting proteins newly identified in this study (Rrp5, Gcy1, Pcs60, Ydl124w and Mdh3), Rrp5p is a known RBP and Mdh3p contains an NAD(P)-binding Rossman fold, a domain that has been previously reported to bind directly to RNA [10], [22]. In contrast, the remaining three (Ydl124w, Gcy1, and Pcs60) do not have identifiable RNA-binding domains. DNA microarray analysis of RNAs enriched in association with TAP-tagged Gcy1 and Pcs60 identified specific RNAs associated with each of these proteins (FDR≤0.01%), but *ASH1* mRNA was not among the targets of either Gcy1 or Pcs60 at a stringent FDR threshold (see section “The novel RBPs confirmed by RIP-Chip…” for further discussion of targets of Gcy1 and Pcs60), consistent with the protein microarray evidence that both proteins are RBPs with broader specificity.

### Mass spectrometry identifies vesicle trafficking proteins associated with yeast mRNA

As a complementary approach we captured poly(A) RNA from cell lysate using oligo(dT) beads, followed by mass spectrometry to identify RNA-associated proteins (Figure 1B). As a control for “background”, we analyzed a sample of unfractionated whole cell lysate by LC-MS/MS, reasoning that proteins detected in this sample would represent potential false positives in the actual mRNA affinity experiment by virtue of being highly abundant. In all cases we considered as significant only those proteins for which two or more unique peptides were identified with a Scaffold protein identification probability of >95% [23].

In each experiment (including the whole cell lysate control) we identified between 83 and 88 proteins that met the above criteria. The proteins identified in the control sample were strongly biased towards abundant proteins (average percentile rank in protein abundance = 97%) [1]; ribosomal proteins (hypergeometric p-value = 9.3×10^−3^), Hsp70-chaperones (hypergeometric p-value = 1.8×10^−6^), and tRNA synthetases (hypergeometric p-value = 2.7×10^−8^) were disproportionately represented in this group. Although many of these highly abundant proteins may indeed bind specifically to RNA, we chose not to investigate them further. 68 proteins identified in the mRNA-affinity sample were not present in the control sample (Table S2). 22 of these 68 proteins were already annotated as RBPs (hypergeometric p-value = 8.8×10^−7^ relative to their representation among mRNAs expressed in these cells). The Gene Ontology (GO) functional categories most significantly enriched among the 68 proteins were related to post-transcriptional regulation: “stress granule assembly” (hypergeometric p-value = 2.4×10^−5^), “mRNA catabolic process” (hypergeometric p-value = 4.1×10^−5^) and “mRNA P-body assembly” (hypergeometric p-value = 1.8×10^−3^).

Unexpectedly, several proteins involved in vesicular transport and secretion (Sec1, Sec16, Sec31, Sec26, Sec27, Ubp3, Gvp36, and Lsp1) were among the 46 proteins identified in the mRNA-affinity isolated samples, but not heretofore annotated as RBPs. Sec3p is required for the proper localization of the *ASH1* mRNA, and *ASH1* co-fractionates with ER microsomes in a Sec3- and She2-dependent manner [24]. However, none of the eight vesicle and membrane trafficking proteins we identified have been implicated in RNA transport or localization. The Sec proteins found by our mass spectrometry approach include components of COPI and COPII, localize to different parts of the cell (early and late Golgi vesicles, ER, bud neck, cell cortex), and are implicated in different stages of vesicle formation and trafficking. Microarray analysis of RNAs associated with Ubp3, a protease with a role in anterograde and retrograde transport between the ER and the Golgi, showed specific enrichment of mRNAs encoding membrane, bud, and cell wall components (see section “Novel RBPs are functionally diverse…”). These results suggest the possibility that the cell's vesicular transport system may play a more significant role in mRNA transport and localization than previously recognized.

Although this method appears to have identified new RNA binding proteins, it is subject to the limitations of conventional mass spectrometry. In each run only ∼85 proteins were identified with high confidence, with a bias towards highly abundant proteins (ribosomal proteins and chaperones). The protein microarray approach therefore appears better suited for a systematic interrogation of the RNA interaction propensity of thousands of different yeast proteins in one rapid experiment.

### Protein microarrays identify both known and unexpected protein-mRNA interactions

To search for novel RNA-protein interactions, we probed protein microarrays with fluorescently labeled yeast total mRNA. The fluorescent intensity of a spot was used as a proxy for the amount of mRNA bound and proteins were ranked by the ratio of normalized mean signal intensity to median background (Table S3). Results of dye swap control experiments correlated well (Spearman correlation coefficient of fluorescence intensity values between mRNA-Cy5 and mRNA-Cy3 experiments = 0.7; data for Cy3-mRNA experiments not shown).

We looked for evidence of artifactual signal variation attributable to variable amount of protein per spot, protein size or charge. We found no relationship between the fluorescent signal from each spot and either the molecular weight (Figure S1 with data from [1]) or the charge of the respective protein (Figure S2 with data from [8]), suggesting that protein charge and size did not introduce a bias in the efficiency of immobilization on the nitrocellulose microarray surface or in the overall affinity of a protein for RNA. We used a fluorescent anti-GST antibody to compare the amount of GST tagged protein present in each spot and found that the relative amount of GST-tagged protein present per spot did not correlate with the amount of fluorescent mRNA bound (Pearson Correlation Coefficient = −0.023), suggesting that our results were not biased by variation in the amount of protein per spot (Figure S3).

**Figure 2:**
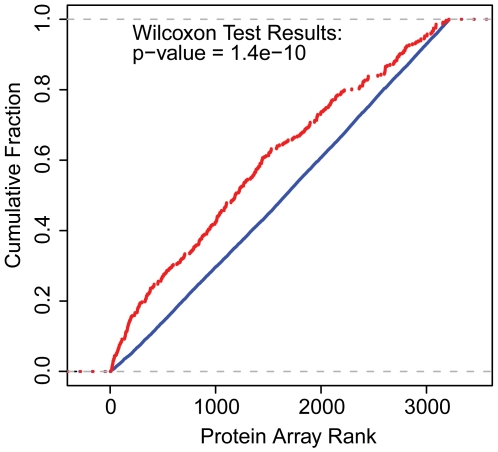
Enrichment of annotated RBPs in the protein microarray data. The red line represents proteins annotated as RBPs, the blue line represents proteins not annotated as RBPs. Protein microarrays were probed with 500 nM polyA-selected RNA labeled with Cy5. Proteins were ranked by the ratio of normalized mean Cy5 signal to median microarray background. The Wilcoxon rank test was used to evaluate the significance of enrichment of known RBPs relative to proteins not annotated as RBPs.

The fluorescent signal representing capture of mRNA was systematically higher for known RBPs than for other proteins (Wilcoxon test p-value = 1.4×10^−10^, Figure 2), suggesting that other proteins with high fluorescent signal might represent novel RBPs. To test this possibility, we used an independent experimental method to identify which, if any cellular mRNAs might be associated with a sample of the novel candidate RBPs.

### Many of the candidate novel RBPs interact with specific RNAs

We selected 35 proteins solely based on their ranks (ranging from 3 to 3,746) in signal intensity in the protein microarray experiments, irrespective of any known function or characteristics of the proteins themselves (Table S4). The 35 sampled proteins included enzymes, vesicle trafficking and intracellular transporters, nucleic-acid binding proteins, actin related/associated proteins, stress-response chaperones, chromatin remodeling components, and methyltransferases. We analyzed each of them by affinity isolation of the protein followed by microarray profiling of any associated RNAs [25] using strains from the yeast TAP-tagged collection. We included two known RBPs (Gus1 and Scd6) as positive controls. Eight “Mock” IPs using lysates from isogenic untagged strains were included as negative controls. We initially performed one IP for each of the 35 candidate RBPs, followed by an additional two replicates of the most promising candidates.

We used a normalization and background subtraction method, described in Materials and Methods, to minimize the contribution of nonspecific background, as modeled by the “Mock” enrichment data. We used the Significance Analysis of Microarrays (SAM) algorithm [26] to compare the replicate assays of each tested protein to the Mock IP results. We considered sets of mRNAs that had a False Discovery Rate (FDR) less than or equal to 0.01% as estimated by the SAM algorithm; in the case of proteins with greater than 500 mRNA targets according to these criteria, we also required a Mock-Corrected Log2 Ratio value greater than 1 (see “Materials and Methods” for detailed explanation).

Based on this analysis, we found that the two “known RBP” positive controls (Gus1 and Scd6) and 12 (34%) of the 35 novel candidates interacted reproducibly with specific sets of RNAs. Some of the candidate RBPs (Lys1, Ubp3, Crg1, Arf3, Pcs60) co-purified with many different, highly enriched transcripts, confirming that these proteins clearly interact with RNA. Other candidate RBPs (Vtc1, Arc15, Hsp26, Arp8, Gis2) co-purified with smaller sets of mRNAs, but these small sets of putative mRNA targets shared distinct functional and/or cytotopical themes, increasing our confidence that the RNA-protein interactions we observed were genuine. Three of the candidate RBPs (Gcy1, Pad1, Bub1) had fewer than 100 targets. Two additional proteins (Smy1, Mtq2) had no targets at a stringent FDR threshold of 0.01%, though they did have putative RNA targets at an FDR of <1%. To empirically test the false positive rate of our analysis procedure, we repeated the analyses, treating randomly selected Mock IP data sets as if they represented results for a candidate RBP (see “Materials and Methods”). In no case was a single mRNA identified as a target of a “fake” RBP (at an FDR <20%), further evidence that our analysis methods are sufficiently stringent. Since Bub1 was specifically associated only with its own mRNA transcript, we do not count it as a *bona fide* RBP. To keep the criteria for identifying novel RBPs conservative, we have not considered Smy1 or Mtq2 in our tally of RBPs, but we report their putative targets for informational purposes (Table S5).

A potential complication of any protein purification is contamination with proteins that co-purify with the protein of interest. When we analyzed the proteins that have been reported to co-purify with the 12 candidate novel RBPs [27], we found that only one has been reported to associate with a known RNA binding protein: Ubp3 forms a complex with the RRM (RNA Recognition Motif)-containing protein, Bre5. Since Ubp3 and Bre5 physically interact to co-regulate the anterograde and retrograde transport between the endoplasmic reticulum and Golgi compartments [28], it is possible that Ubp3-Bre5 may also bind to RNA as a complex. None of the other candidate novel RBPs has been reported to co-purify with any known RNA binding proteins; contamination with co-purifying proteins is therefore unlikely to account for these results.

While our overall validation rate was 34%, proteins with greater signal intensity/background in the protein microarray experiment were significantly more likely to be validated by our IP microarray experiments (Kolmogorov-Smirnov test p-value = 0.03). Six of the eight proteins tested that ranked among the top 50 in signal intensity in the protein microarray experiments co-purified with specific sets of RNAs. 26 additional proteins that ranked in the top 50 are not currently annotated as RBPs; these represent good candidates for investigation by IP microarray experiments (Table S6).

### Novel RBPs are functionally diverse and lack recognizable RNA-binding domains

Mirroring the variety of functional classes that were initially included in the IP experiments, the proteins that we found associated with RNA *in vivo* carry out diverse functions and localize to different sub-cellular compartments (Table 2). Lys1 and Gis2 are the only proteins that contain domains known to bind to nucleic acid. Lys1 contains an NAD(P)-binding Rossman fold domain motif similar to the one reported to have RNA-binding activity in GAPDH [10], and Gis2 is a zinc-finger domain containing protein (independently identified as an RBP by another group (Scherrer and Gerber, submitted). The remaining RNA-binding proteins newly identified in this study do not have domains known to bind nucleic acids.

**Table 2:** Novel RBPs identified in the protein microarray experiments and confirmed by IP-microarray experiments.

Name	Function/Description	Number of Targets (at FDR≤0.01)	Cellular Component
**Scd6**	**RNA processing**	**1711**	**Cytoplasm**
**Gus1**	**t-RNA synthetase**	**12**	**Cytoplasm, mitochondrion**
Arc15	Motility of actin patches	171	Actin patch, mitochondrial outer membrane
Arf3	Ras-GTPase; patch localization	855	Bud neck, bud tip
Arp8	Actin-related; chromatin remodeling	231	Nucleus
Crg1	Putative SAM-dependent methyltransferase	419	Unknown
Gcy1	NADP+- dehydrogenase; glycerol catabolism	44	Cytoplasm, nucleus
Gis2	Zn-finger; Ras signaling	151	Cytoplasm
Hsp26	Chaperone; heat shock response	279	Nucleus, cytoplasm
Lys1	NAD+- dehydrogenase; lysine biosynthesis	1353	Cytoplasm
Mtq2	SAM-dependent methyltransferase	111 *	Cytoplasm, nucleus
Pad1	Decarboxylase	86	Mitochondrion
Pcs60	AMP-binding synthetase; fatty acid metabolism	969	Cytoplasm, peroxisomal membrane/matrix
Smy1	Exocytosis; kinesin	152 *	Mating projection tip
Ubp3	Protease; ER-to-Golgi transport	1110	Cytoplasm
Vtc1	Chaperone; trafficking	243	ER, vacuolar membrane

For each of these RBPs, three replicate IP-microarray experiments were performed. In bold, are shown predicted and known RBPs that were used as positive controls. Number of targets with FDR≤0.01% (determined using SAM) is shown for each protein. Smy1 and Mtq2 (*) is an exception- it has no targets at FDR≤0.01% and we used FDR≤1% as a cut-off. Protein localization data from [8].

**Figure 3:**
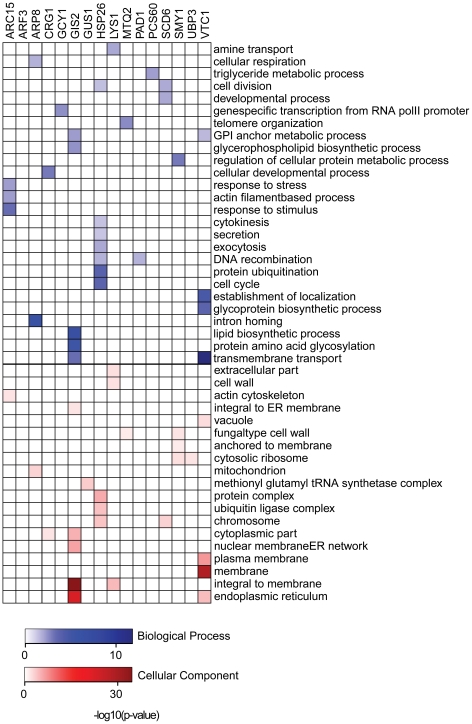
GO Term enrichment of targets for novel RBPs identified by IP-microarrays. Enrichment was determined using GO Term search by [43] on target genes with FDR≤0.01% (with the exception of Smy1 and Mtq2, which are not counted as RBPs, for which targets with FDR≤1% were considered). Hypergeometric p-value<0.05 (corrected for multiple hypothesis testing) was used as a cut-off. Complete target datasets are available in Table S5. In blue, are shown enriched “Biological Process” and in red- “Cellular Component”.

One interesting example of a novel RBP lacking a known RNA binding domain is Crg1- a putative S-adenosyl-methionine methyltransferase, whose targets are enriched for mRNAs encoding proteins important for cellular developmental processes like the maintenance of chromosome integrity (Table S5 and Figure 3). The substrates methylated by diverse SAM methyltransferases include DNA, rRNA, proteins and small molecules and many methyltransferases are involved in transcriptional control ([29] and others) and receptor-mediated signaling [30]. In addition, histone methyltransferases have been found in a complex with proteins and mRNAs [31]. However, to the best of our knowledge, Crg1 is the first example of an S-adenosyl-methionine methyltransferase that associates with mRNA. One possibility is that Crg1 may methylate its target mRNAs, potentially as a means of regulating their translation or stability. Alternatively, the methyltransferase target RNAs could guide the enzyme to specific loci, a model recently proposed by Khalil et al. for mammalian lincRNAs and chromatin-modifying components [32]. Another possibility in light of recent reports [31] is that Crg1 and its target mRNAs are recruited to the same site to facilitate localized translation and protein complex assembly.

Other novel RBPs appear to play dual roles as metabolic enzymes and RNA-binding proteins; Lys1, Gcy1, and Pcs60 encode enzymes that participate in lysine biosynthesis, glycerol and fatty-acid metabolism, respectively. Pcs60 co-purified with mRNAs encoding proteins involved in triglyceride metabolism (hypergeometric p-value = 8.5×10^−3^) (Table S5 and Figure 3). Lys1 interacted with >1,000 mRNAs, of which the most highly enriched include a remarkably high fraction intrinsic membrane proteins (hypergeometric p-value = 1.4×10^−7^), particularly amino acid transporters (hypergeometric p-value = 1.0×10^−2^) (Figure 3).

Arc15- a component of the Arp2/3 complex- co-purified with the *LAS17* mRNA, which encodes the assembly factor of the Arp2/3 complex, as well as 10 other mRNAs encoding components of the actin patch organization (Table S5 and Figure 3). It is possible therefore that the co-localization of these mRNAs could subsequently direct their localized translation and promote the formation of the Arp2/3 protein complex and the assembly of the actin patch.

Vtc1 and Ubp3 are involved in vesicular transport [33], [34]. Vtc1 preferentially associated with transcripts encoding ER, membrane, and vacuolar components (Figure 3). Ubp3 had >1,000 targets at an FDR threshold of ≤0.01%. Among the Ubp3 targets, we observed enrichment of mRNAs encoding protein components of the cell cortex (Kolmogorov-Smirnov test p-value = 2.5×10^−3^), cellular bud (Kolmogorov-Smirnov test p-value = 7.1×10^−4^), and plasma membrane (Kolmogorov-Smirnov test p-value = 2.5×10^−3^). Since Ubp3 physically interacts with the known RBP, Bre5, it is possible that the apparent RNA-binding activity of Ubp3 is attributable to Bre5. Our mass spectrometry results identified an 7 additional vesicle and membrane trafficking proteins (components of both COPI and COPII- Table S2) that could also represent *bona fide* RBPs, though we have yet to confirm them independently. The significant enrichment of mRNAs encoding proteins found in specific sub-cellular compartments among the targets of the transport/trafficking proteins Vtc1 and Ubp3 suggests that proteins with distinct roles in trafficking membranes may also participate in the transport and localization of mRNAs to specific sub-cellular locations.

The two positive control RBPs, Scd6 and Gus1, immunopurified with RNA as expected (Table S5 and Figure 3). Interestingly, the RNAs most enriched in association with Gus1 were *MES1* and *ARC1*, encoding two proteins with which Gus1 forms a complex *in vivo* [35]. It was therefore plausible that the association between Gus1 and the *ARC1/MES1* mRNAs might be mediated by co-translational assembly of a complex of the three proteins [31]. We tested this hypothesis by isolating Gus1 in the presence of EDTA to disassemble ribosomal subunits and disrupt any linkage between a nascent polypeptide and its cognate mRNA. EDTA did not disturb the association between the Gus1 protein and the *ARC1-MES1* mRNAs (data not shown). These results are consistent with a scenario in which *ARC1* and *MES1* are co-localized independent of translation by direct interaction with Gus1—potentially as a means of coordinating the translation and subsequent assembly of the Gus1-Arc1-Mes1 protein complex.

In summary, our follow-up IP-microarray experiments revealed RBPs among several unexpected classes of proteins, most with known ”day jobs” as enzymes in metabolic and other processes. In addition, we found evidence for the coordinated regulation of mRNAs encoding subunits of protein complexes and a possible link between mRNA trafficking and vesicle transport. Our results add to the growing number of enzymes that appear to bind specific RNAs and may have roles in post-transcriptional regulation ([10], [36], [37], [38] and others), despite the lack of conventional RNA-binding domains. Their unexpected identification by this empirical approach highlights the value of reliable, scalable experimental methods to look for novel RBPs.

### Summary

Accumulating evidence points to a vast network of RNA-protein interactions in yeast, mammals and other eukaryotes, but the features and molecular components of this network are still largely unexplored. Much of what is known about the ∼600 annotated RNA binding proteins in *Saccharomyces cerevisiae* suggests that there are more RBPs to be identified, many among unexpected classes of proteins lacking known RNA binding domains. In light of the limitations of current bioinformatic methods that rely upon homology to known RNA binding domains, there is a clear need for systematic scalable experimental methods for the discovery of novel RBPs.

We developed two experimental approaches to search for novel RNA-protein interactions. One method combines affinity purification with mass spectrometry to identify proteins from a whole cell lysate that co-purify with total mRNA. This method yielded interesting novel RBPs (Table S2), but is subject to the limitations of conventional mass spectrometry. It identified only a relatively small number of proteins, significantly biased towards highly abundant proteins. Consequently, we developed a more robust approach that uses protein microarrays and overcomes the limitations of mass spectrometry. We designed microarrays that represent >80% of the yeast proteome, enabling a nearly genome-wide interrogation of RNA-protein interactions *in vitro*. While we focused here on how this method can be used to search for novel RNA binding proteins, the protein microarray platform has other potential applications, including discovery of novel RNA-protein interactions for an individual RNA or a defined pool of RNAs. As an illustration, we successfully employed the protein microarrays to look for proteins that interact specifically with the *ASH1* mRNA. Protein microarrays could also be used to detect selective RNA-protein interactions with modified (methylated, etc.) or partially processed (spliced, capped, etc.) mRNAs.

Our systematic search for novel RBPs yielded several unexpected findings. Data from the MS and protein microarray approaches confirmed that there are still many more RBPs than have been recognized to date. In follow-up experiments, 12 of 35 selected candidate novel RBPs with a range of ranks in the protein microarray data co-purified with specific sets of mRNAs with shared functional themes. A majority of the 12 novel RNA-binding proteins were enzymes, adding to growing evidence that enzymes can have important RNA-binding roles ([10], [39] and others). With the exception of Gis2 and Lys1, the novel RBPs described in this work have no known RNA binding domains, suggesting the possibility that the evolution of these RNA-protein interactions involved selection for RNA aptamer-like elements in the mRNAs, capable of binding selectively to a protein; i.e. the RNAs evolved protein-specific domains and not vice versa. In support of this hypothesis, aconitase, originally annotated as an enzyme with a central role in energy production, was later discovered to play a role in post-transcriptional regulation of iron metabolism through interaction with an RNA stem-loop aptamer [40], [41]. Recent work has shown that a conformational switch to form a tRNA aptamer is at the heart of the mitochondrial import of specific tRNAs by enolase-preMSK1 [11]. Additional experiments will be required to define the structural basis of the RNA-protein interactions and their evolutionary origins, and to determine exactly how the fate of target mRNAs is affected by the novel RBPs.

Following the law of parsimony, individual proteins are generally assumed to have a single function. There is increasing evidence, however, that many proteins have more than one important role; RNA-binding may be a particularly common “moonlighting” role. Searching for RNA-binding activity in unlikely places requires an efficient, scalable experimental approach. The two genome-wide experimental methods presented here may help to shed light on the largely unexplored landscape of post-transcriptional regulation. Our results suggest that the cell's network of RNA-protein interactions is larger and richer than expected. An analogous study should enable unexpected RBPs to be discovered in humans and other organisms of interest.

## Materials and Methods

### Purification of GST-tagged ORFs

Glycerol stocks were streaked on SC-Ura plates (6.7 g Difco Yeast Nitrogen Base without Amino Acids (Difco Cat# 291940), 2 g SC-Ura (Sunrise Scientific Cat# 1306–030), 20% glucose, 20 g agar) and colonies were inoculated in SC-Ura liquid medium (6.7 g Difco Yeast Nitrogen Base without Amino Acids, 2 g SC-Ura, 20% glucose). The liquid cultures were allowed to grow overnight, then 100 uL of each were diluted in 2 mL of SC-Ura media with 2% raffinose (6.7 g Difco Yeast Nitrogen Base without Amino Acids, 2 g SC-Ura, 20% raffinose) and cultures were grown to OD600 ∼1.0. Then, expression of the protein was induced by the addition of 200 uL of 20% galactose (to a final concentration of 4%) followed by growth for 6 hours with shaking. Half of each culture was added to a 96-well plate and pelleted by centrifugation at 3,000rpm for 3 minutes at 4°C. The remaining half of each culture was added and the centrifugation step was repeated. Then, 250 uL of acid-washed glass beads were added to each well and the cells were frozen at −80°C.

On the day of lysis, 50 uL of Tris Lysis I Buffer (50 mM Tris-HCl pH 8.0; 150 mM NaCl; 5 mM EDTA; 5% glycerol; Roche protease inhibitor complete; 1 mM PMSF; 0.5 mM DTT) and cells were lysed in a Harbil paint shaker (Fluid Menagement) for 3 cycles of 2 minutes each, placing the plates on ice for 2 minutes in between cycles. Then, the plates were centrifuged 3,000rpm for 1 minute at 4°C and 600 uL of Tris Lysis II Buffer (50 mM Tris-HCl pH 8.0; 150 mM NaCl; 0.5 mM EDTA; 5% glycerol; 1% Triton X-100; 1 mM PMSF; 0.5 mM DTT) was added to each well. Cells were lysed 2 additional times for 2 minutes each in the paint shaker and centrifuged at 3,000rpm for 10 minutes at 4°C. In the meantime, glutathione-sepharose 4B beads (Amersham Cat#17-0756-01) were washed with 1 bed volume of Tris Lysis II Buffer 3 times, spinning at 3,000rpm for 1 minute at 4C to collect beads between washes. Beads were resuspended in Tris Lysis II Buffer to yield 50% slurry. 600ul of the spun-down lysate were transferred to a clean 1.2 ml 96-well plate and 75ul of equilibrated 50% glutathione-sepharose were added to each well. Plates were incubated at 4°C for 2 hours with shaking (200rpm), inverting manually 1–2 times during the 2-hour interval to ensure mixing. Then, the plates were spun down at 3,000rpm for 2 minutes at 4°C and the supernatant was discarded. The bead slurry was transferred to a 1.2 micron 96-well PVDF filter-plate (Millipore) sealed over a polypropylene receiving plate and spun at 3,000rpm for 1 minute at 4°C. The filtrate was discarded and the beads were washed three times with 200 uL Hepes Wash Buffer (50 mM Hepes pH 7.5; 300 mM NaCl; 5% glycerol; 0.1% Triton X-100; 0.1 mM PMSF; 0.5 mM DTT) each; centrifuging 3,000rpm for 1 minute at 4°C in between washes. 50 uL of Elution Buffer (50 mM Hepes pH 7.5; 200 mM NaCl; 25% glycerol; 0.08% Triton X-100; 0.5 mM DTT; 20 mM reduced glutathione; sodium hydroxide to a pH of 7.5–8.0) were added to each well and the PVDF/receiving plates were sealed and incubated at 4°C overnight. On the next morning, the plates were centrifuged 3,000rpm for 10 minutes at 4°C, the PVDF filter plate was discarded and the receiving plate (containing the eluted protein) was sealed and frozen at −80°C.

### Protein microarray design

We purified >4,700 proteins from the yeast GST-tagged collection (Open Biosystems Cat# YSC4423). Arrays were printed on modified nitrocellulose coated glass PATH slides (GenTel Biosciences Cat# 2-1025) with a 48-pin contact printer (Bio-Rad ChipWriter Pro). The surface of these PATH slides is optimized for printing protein microarrays and designed for applications that require fluorescent detection [42]. We also included spots containing elution buffer only for the purpose of ruling out background fluorescent signal due to the composition of the elution buffer alone. Also, Cy3-anti-biotin antibody spots were also printed in each corner and used for proper alignment of the blocks.

In order to estimate amount of GST-protein per spot, we probed arrays with monoclonal anti-GST conjugated to Hilyte Fluor 647 (Abcam Cat# ab64370) at 1∶50 dilution. Arrays were blocked, probed and processed as described in the section below. Proteins were ranked based on mean Cy5 of a spot divided by normalized Cy5 background signal as a proxy for amount of protein present in each spot. No correlation was found between this ranking and the ranking of proteins based on affinity for total mRNA (data not shown).

### Protein Microarray sample preparation and hybridization

Total RNA was extracted from mid-log yeast grown in YPD media using PureLink Micro-to-Midi Kit (Invitrogen Cat# 12183-018). PolyA-RNA was selected and amplified with Ambion's Aminoallyl MessageAmp II aRNA Kit (Ambion Cat# AM1753) to make antisense-RNA, which was tailed and used for a second round of selection and amplification with the kit to make mRNA. The mRNA concentration was determined using a Nanodrop.

*ASH1* was PCR-amplified using the following primer pair: forward 5′-CGAGCTCATGTCAAGCTTATACATCA-3′ and reverse 5′-CGATATCTCAATTCTCTACTGTCT-3′. The DNA product was digested with SacI and EcoRV and cloned into a pBluescriptII KS+ phagemid (Stratagene Cat# 212207) and the sequence of the DNA was confirmed by Sanger sequencing prior to *in vitro* transcription. The *ASH1* RNA yield was quantified with a Nanodrop and size was verified by running on a denaturing gel.

Prior to microarray hybridization, each microarray was allowed to equilibrate at 4C for at least 15 min and then pre-blocked in 25–30 mL Blocking Buffer (1X PBS; 1% BSA w/v; 1 mM DTT; 50 µg/mL E.coli tRNA; 20 µg/mL heparin) at 4°C for 2 hours with gentle shaking. For the RBP discovery experiments, 10 µg of poly(A)-selected mRNA and 60 picomoles of specific *in vitro* transcribed control RNA were labeled with Cy5 and Cy3-dye NHS-monoesters (GE Healthcare Life Sciences Cat# RPN5661) respectively. For the *ASH1* experiments, 60 picomoles of *in vitro* transcribed *ASH1* mRNA and 10 µg of total mRNA were labeled with Cy5 and Cy3, respectively. For dye-swap experiments, dyes were reversed. Excess dye was removed using Ambion's AminoAllyl MessageAmp II aRNA Kit (Ambion Cat# AM1753) by pooling the Cy5 and Cy3 labeled samples before loading on the clean-up columns. The labeled pool was eluted twice in 20 ul of preheated water each and dye incorporation was quantified using a Nanodrop.

A total of 30 uL of eluted RNA sample was mixed with 30 uL of 2X Sample Buffer (40 mM Tris-HCl pH 8.0; 150 mM NaCl; 4 mM MgCl2; 10% glycerol; 0.1% Triton X-100; 2% BSA w/v; 2 mM DTT; 40 µg/mL heparin; 0.4 mg/mL E.coli tRNA) and 0.2 uL of Superase-IN and pipetted on a protein chip. The microarray was incubated for 90 minutes at RT in the dark. Each slide was washed twice for 10 minutes at 4°C each with ∼25 mL cold 1X Sample Buffer (with 5 U/mL Superase-IN) and twice for 10 minutes at 4°C each with ∼25 mL cold 1X Sample Buffer (with 5 U/mL Superase-IN) without E.coli tRNA. The microarray was dried in an ozone-free centrifuge by spinning at 300rpm for 4 minutes at room temperature and scanned using either Axonscanner 4000A or 4000B (Molecular Devices). The intensity of each protein spot was analyzed with the GenePix Pro 6.0 software (Molecular Devices). Microarray data were uploaded on the Stanford Microarray Database (http://smd.stanford.edu/cgi-bin/login.pl) and are available for download. Proteins were ranked based on mean-normalized channel intensity/normalized background. For identifying specific *ASH1*-interactors, mean-normalized Log2(*ASH1-*Cy dye)/(total mRNA-Cy dye) ratios were calculated. Enriched GO Terms were identified using SGD's GO Term Finder (http://www.yeastgenome.org/cgi-bin/GO/goTermFinder.pl). To calculate the RBP enrichment threshold, we created a sliding window plot (window size of 200) to plot the fraction of annotated RBPs vs. the protein microarray signal-to-background (Figure S4). We then found the RBP enrichment threshold as the point at which this line first equals the average frequency of annotated RBPs in the entire data set (0.11), which is at a signal-to-background value of 1.65 (and a relative rank of ∼480). This threshold was later used to calculate the significance of the relationship between protein microarray rank and the candidate novel RBP validation rate.

### mRNA Pulldown-LC-MS/MS Assay

For the mRNA pulldown experiments, we used BcMag®mRNA oligo(dT) beads from Bioclone (Cat# MMS-106) with binding capacity 1 ml beads per 2mgs total RNA. Beads were equilibrated in Binding Buffer (100 mM Hepes pH 7.5; 500 mM LiCl; 10 mM EDTA; 10 mM DTT; Superase-IN). We compared the efficiency of capture of a known RBP when oligo(dT) beads were preincubated with total RNA isolated from exponentially growing yeast prior to addition of whole cell lysate (run #1) to that when oligo(dT) beads were directly incubated with the lysate (run #2). There was no apparent difference between the two approaches in the amount of one specific RBP (Ypl184c) that was recovered in each case monitored on a Western blot (data not shown). In run #1, we preincubated beads with 2mgs of total yeast RNA, isolated with Purelink Micro-to-Midi kit (Invitrogen Cat#12183-018) from mid-log phase cells in YPD, for 10 minutes at room temperature on a rotator and then added whole cell lysate (also from mid-log phase cells) and incubated for another 10 minutes at room temperature on a rotator. In run #2, we added yeast lysate from cells in mid-log phase in YPD directly to the beads and incubated for 10 minutes at room temperature on a rotator. After incubation with lysate, the beads were washed 4 times each in four volumes of Washing Buffer (10 mM HEPES pH 7.5; 150 mM LiCl; 1 mM EDTA) and RNA-protein complexes were eluted by heating at 65°C for 3 minutes in 1 mL of Elution Buffer (10 mM Hepes pH 7.5). Eluate was concentrated and submitted for in-solution tryptic digest and LC-MS/MS analysis at the Stanford University Mass Spectometry Facility (http://mass-spec.stanford.edu/). Data were analyzed using Scaffold Proteome Software (http://www.proteomesoftware.com/proteome_software_scaffold_sample_data.html). Enriched functional categories were identified using SMD's GO Term Finder and hypergeometric p-values were corrected for multiple hypothesis testing [43].

### Purification of Candidate RNA-Binding Proteins and Associated RNAs

Candidate RNA-Binding Proteins (RBPs) were affinity purified and their associated RNAs were identified by microarray analysis, essentially as previously described [5]. A total of 35 candidate RBPs (Table S4) were selected for validation based on their relative rank in the protein microarray data (ranked by signal/background) alone. The protein microarray ranks of the candidate RBPs that were selected for validation varied widely, from 3 to 3,746. Initially, 1–2 replicate affinity purifications were performed for each candidate RBP. TAP-tagged yeast strains derived from BY4741 (Open Biosystems Cat# YSC1177) were grown to an OD_600_ of 0.6–0.8 in minimal media (6.7 g Difco Yeast Nitrogen Base without amino acids, 60 mg L-Leucine, 20 mg L-Histidine, 20 mg L-Methionine, 20 mg Uracil, and 20 g glucose per liter) or YPD [1]. For each IP, cells from mid-log phase cells were harvested by centrifugation, washed twice with buffer A (50 mM HEPES pH 8.0, 140 mM KCl, 1.8 mM MgCl2, 0.1% NP-40, and 0.2 mg/mL heparin), resuspended in buffer B (buffer A with 1ug/mL Pepstatin, Leupeptin, and Vanadate, 2.5ug/mL Aprotinin, 1 mM PMSF, 0.5 mM DTT, and 0.1U/uL Superasin Rnase inhibitor from Ambion), and lysed by Mini bead-beater 8 from Biospec products (Cat# 693) with four 1-min cycles at max speed. Lysate was cleared by centrifugation for 10 minutes at 8,000xg and 4°C, and total protein concentration was adjusted to ∼15 mg/mL by dilution with buffer B. Biotinylated rabbit IgG was coupled to streptavidin coated magnetic beads (Invitrogen Cat# 602-10). Beads were incubated with lysate for 2 hours, then washed for 15 minutes on rotator at 4°C, once with buffer B and three times with buffer C (buffer B with 10% Glycerol and no heparin or vanadate). 100uL of the lysate remaining after the beads were removed was set aside for the isolation of reference RNA. IP RNA was isolated with phenol:chloroform as described elsewhere [5]. Total RNA for use as a reference was purified from the lysate remaining after the 2 hour incubation with the beads, using PureLink Micro-to-Midi Kit (Invitrogen Cat# 12183-018).

For the initial round of IPs, a total of four separate negative control purifications (“Mocks”) done with lysate from untagged BY4741 strains were performed on cells grown in either minimal media or YPD (two with each media type). Candidate RBPs from cells grown under different media conditions were purified separately, and the microarray data was analyzed separately. Proteins that co-IPed with RNAs very different than the Mocks were grown in minimal media and re-purified in duplicate as described above. For the second round of experiments, a total of 6 mock IPs were performed. This yielded a total of 2–3 replicates for the most promising candidate RBPs, and 8 mock IP replicates.

### DNA Microarray Production and Pre-hybridization Processing

Yeast DNA microarrays were printed on epoxysilane-coated glass (Schott Nexterion E) by the Stanford Functional Genomic Facility. The DNA oligonucleotide printed were previously described [5]. Further information about the probes used, including probe sequences, is available from the Operon Web site (https://www.operon.com/; S. cerevisiae YBOX V1.0).

Detailed protocols for microarray experiments can be found on the Brown lab website (http://cmgm.stanford.edu/pbrown/protocols/index.html). The microarray prehybridization performed has been previously described [44]. Within 24 hours prior to hybridization, slides were placed in a humidity chamber (Sigma Cat# H6644) filled with 100 mL of 0.5× SSC (1× SSC = 150 mM NaCl, 15 mM sodium citrate [pH 7.0]) for 30 minutes at room temperature. Slides were then dried rapidly at 70–80°C on a heat block. The epoxysilane surface of the slides was blocked by incubation with 1M Tris-HCl (pH 9.0), 100 mM ethanolamine, and 0.1% SDS for 20 min at 50°C. After blocking, the slides were washed twice for 1 min with 400 ml of water, and then dried by centrifugation.

### DNA Microarray Sample Preparation, Hybridization, and Washing

PolyA-RNA was selected, amplified, purified, and labeled with Cy-dyes using Ambion's Aminoallyl MessageAmp II-96 aRNA Kit (Ambion Cat# AM1819). Up to 5 µg of RNA was used as input for each RT reaction. In-vitro transcribed RNA was then coupled to NHS-monoesters of either Cy5-dye for RNA that co-purified with the candidate RBP, or Cy3-dye for the reference RNA (GE Healthcare Life Sciences Cat# RPN5661).

Up to 10ug of Cy5-labeled samples were pooled with 10ug of their appropriate Cy3-labeled counterparts, and combined with the components of Hybridization Buffer A (3× SSC, 25 mM Hepes-NaOH (pH 7.0), 20 µg of poly(A) RNA (Sigma Cat# P9403), and 0.3% SDS) to yield a total volume of 50uL. Samples were then heated to 70°C for 5 minutes, spun at 14,000 rpm at room temperature in a microfuge for 10 minutes, then hybridized at 65°C using the MAUI hybridization system (BioMicro) for 12–16 h.

After hybridization, slides were washed first in a solution of 2x SSC with 0.05% SDS at 70°C for 5 minutes, then in 2x SSC at room temperature for 2 minutes, then in 1x SSC at room temperature for 2 minutes, then 0.2x SSC at room temperature for 2 minutes. Slides were then dried dried by centrifugation in a low-ozone room (<5ppb).

### DNA Microarray Scanning and Data Processing

Microarrays were scanned using AxonScanner 4000B (Molecular Devices). PMTs were adjusted to maximize signal, without excessive background and pixel saturation. Microarray spots were located and their data extracted using the GenePix Pro 6.0 software (Molecular Devices). All data is MIAME compliant and the raw data has been deposited in a MIAME compliant database. The microarray data have been submitted to Stanford Microarray Database (http://smd.stanford.edu/cgi-bin/login.pl) and Gene Expression Omnibus (GEO) (www.ncbi.nlm.nih.gov/geo/) under the accession number GSE22876. The data was filtered for signal vs. background using several parameters. Specifically, the Cy5 (red) vs Cy3 (green) pixel intensity values for each spot must have a correlation coefficient (R-squared) >0.6. In addition, the signal intensity minus the local background for each spot must be greater than 100, or greater than 3x the standard deviation of the local background (surrounding each spot). Signal in either channel that failed these filtering criteria was considered absent. Spots with green signal but no red signal were kept separated as RNAs that were expressed but did not co-purify with the candidate RBP. Finally, both the technical replicates of each DNA oligonucleotide (each oligonucleotide was printed twice per microarray) had to pass filtering for that spot to be considered as a possible target of a given candidate RBP. The log (base 2) of the Cy5 to Cy3 ratio (Log2 Ratio or L2R) for each spot that passed filtering was used for the subsequent analyses.

### Analysis of DNA Microarray Data—Part A

The single IP data from our initial list of 35 candidate RBPs were analyzed to determine if a given candidate RBP was co-purifying with a set of RNAs that was significantly different from the RNAs that co-purify with the mock un-tagged control experiments. The purpose of this analysis was to select the most promising candidate RBPs for additional replicate purifications. The method we used is based on the observation that microarray analysis of RNAs enriched in replicate assays of known RBPs or replicates of negative controls (Mocks), respectively, each have high Spearman correlation coefficients between the resulting enrichment ratios, but when enrichment results for a known RBP are compared to those for a Mock experiment, the correlation coefficient tends to be much smaller. The Spearman correlation coefficient between a known RBP and a Mock tends to decrease as the number of targets of that RBP increased.

First, Spearman rank correlation coefficients were calculated on the intersection of the Log2 Ratio data from a given candidate RBP and the average of the Mock replicates. To create a null distribution for the rank correlation coefficient, replicate spots from two Mock microarrays were treated as a separate microarray by enumerating replicate spots 1, 2, 3, or 4 arbitrarily and assigning all spots with “i” to the ith array, resulting in 4 Mock arrays. These Mocks were then permuted by randomizing the microarray names on a gene by gene basis (240,000 possible permuted mock arrays). Then, Spearman rank correlation coefficients were calculated for 60,000 pairwise comparisons between permuted Mocks. These correlation coefficients were to compute an empirical p-value for testing for significantly decreased correlation against the average Mock at a level of p<0.005. Fifteen of the 35 candidate RBPs were significant at this level (without correction for multiple hypothesis testing). For more rigorous validation and testing they were tested again by the IP microarray assay in duplicate, providing 2 additional replicates of each.

### Analysis of DNA Microarray Data—Part B

To identify the specific RNAs that were associated with each candidate RBP, the Log2 ratios from each microarray were normalized to the average of the Mock arrays (the average of the Mock arrays was calculated after median normalization). To do this, we used an algorithm (Salzman and Klass, in preparation). Briefly, the algorithm selects a set of genes (here we used a set size of 450) that have the greatest difference in relative rank (ranked by Log2 ratio) between the Mock and the candidate RBP microarray. These genes are enriched in the Mock, suggesting they are prominently composed of background signal, but they are not enriched in the RBP IP, suggesting they are not strong RBP targets. Therefore, this set of genes is presumed to model true background in both the Mock and RBP IP. Each distribution is normalized so that the mean of the set of selected background genes is the same in each IP and the average Mock. As the distributions have been normalized relative to each other, the contribution of background binding (represented by the Mock) to the Log2 ratio values for an RBP can simply be subtracted on a gene-by-gene basis, yielding Mock corrected Log2 ratios for each gene. Genes with Mock corrected Log2 ratios greater than zero theoretically represent genes that were more enriched in the RBP IP than in the Mock IP, however, by its nature the procedure can produce some false enrichment.

Every microarray in the experiment was processed as described above, including the Mock arrays. Each Mock microarray was normalized relative to the average of all the other Mock arrays, and the average of all the other Mock arrays was then subtracted from it. This resulted in Mock corrected Log2 ratio values for every microarray, including the Mock arrays.

The Mock corrected Log2 ratios for each microarray were then used as input to the Significance Analysis of Microarrays (SAM) algorithm [26]. 100,000 permutations of 2-class SAM analysis were performed comparing all replicates of each RBP to all replicates of the Mock. We took as targets any mRNAs that had a SAM-calculated False Discovery Rate (FDR) less than or equal to 0.01%, and in the case of proteins with many mRNA targets (greater than 500) we also required a Mock Corrected L2R value greater than 1 (Table S5). We called proteins with mRNA targets that met these criteria *bona fide* RBPs. Gene Ontology (GO) term enrichment for the mRNAs associated with each candidate RBP was calculated comparing these mRNA targets to all the genes that had green signal above background on the microarray using the hypergeometric test (Figure 3), correcting for multiple hypothesis testing [43]. To test whether there was a significant relationship between the protein microarray rank and the probability of validation by IP microarray, we used the KS-test on all proteins we had purified with ranks above the RBP enrichment threshold (at a protein microarray rank of ∼480, see Figure S4).

To test the false positive rate of our analysis method, pairs of Mock IPs were removed at random from the set of Mock experiments and these pairs of Mock IPs were submitted to the analysis script as “fake” candidate RBPs only. In this step, the pair of Mock IPs being used as “fake” candidate RBPs was excluded from the calculated Mock distribution. This procedure was repeated with the 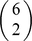 different pairs of Mock IPs, and never produced a single mRNA target of these “fake” candidate RBPs (at an FDR <20%).

## Supporting Information

Table S1 Annotated Saccharomyces cerevisiae RNA-Binding Proteins. Data is compiled based on Gene Ontology information, protein domain homology and gene descriptions from the Saccharomyces Cerevisiae Database (http://www.yeastgenome.org/).
(0.19 MB XLS)

Table S2 Proteins interacting with polyA-mRNA identified by LC-MS/MS in two experiments. We removed proteins identified by LC-MS/MS from whole cell lysate samples alone (“Background”). In “run1”, oligo(dT) beads were preincubated with purified total RNA prior to addition of whole cell lysate. In “run2”, oligo(dT) beads were incubated directly with yeast lysate. Protein abundance data are taken from [1]. Also shown is the rank for each protein from protein microarray experiments with total mRNA (data summarized in Table S3). Indicated by bold text are annotated RBPs (data summarized in Table S1).
(0.03 MB XLS)

Table S3 Summary of protein microarray data. Proteins are ranked according to the affinity for total yeast mRNA (as measured by Cy5/normalized background). Data (“Mean” signal) are averaged for 3 replicate experiments.
(1.18 MB XLS)

Table S4 Proteins chosen for follow-up IP-microarray experiments. These proteins have ranks ranging between 3 and 3746. In bold, are shown proteins also identified by the polyA-mRNA IP- LC-MS/MS experiment (refer to Table S2 for data).
(0.03 MB XLS)

Table S5 mRNA targets of novel RBPs identified in this study. An FDR≤0.01% was used as a cut-off for all proteins, except for Smy1 and Mtq2. Smy1 and Mtq2 were not considered RBPs, but targets are included for informational purposes.
(1.92 MB XLS)

Table S6 Potential novel RBPs based on protein array data. Proteins with ranks 1–50 in protein microarray data that were not included in follow-up IP-microarray experiments and could represent novel RNA-binding proteins.
(0.03 MB XLS)

Figure S1 No bias based on protein size found in protein microarray data. Data for protein weight from [1].
(3.24 MB TIF)

Figure S2 No bias based on protein charge found in protein microarray data. Data for protein weight from [2].
(3.41 MB TIF)

Figure S3 No bias based on amount of protein per spot found in protein microarray data. Amount of protein was estimated based on signal intensity of Hilyte Fluor 647-conjugated anti-GST antibody (1∶50) the protein arrays were probed with.
(3.40 MB TIF)

Figure S4 RBP Enrichment threshold for protein microarray data. A sliding window plot of 200 proteins is used. The gray line indicates the microarray signal-to-background value, at which the average frequency of annotated RBPs for the entire microarray is reached ( = 0.11), and the protein microarray data are not significantly enriching for RBPs.
(1.79 MB TIF)
