# Supplementary figures and images for: Proteome-Wide Search Reveals Unexpected RNA-Binding Proteins in Saccharomyces cerevisiae
Source: PLoS One. 2010 Sep 10;5(9):e12671. doi: 10.1371/journal.pone.0012671 (PMC2937035; doi:10.1371/journal.pone.0012671)

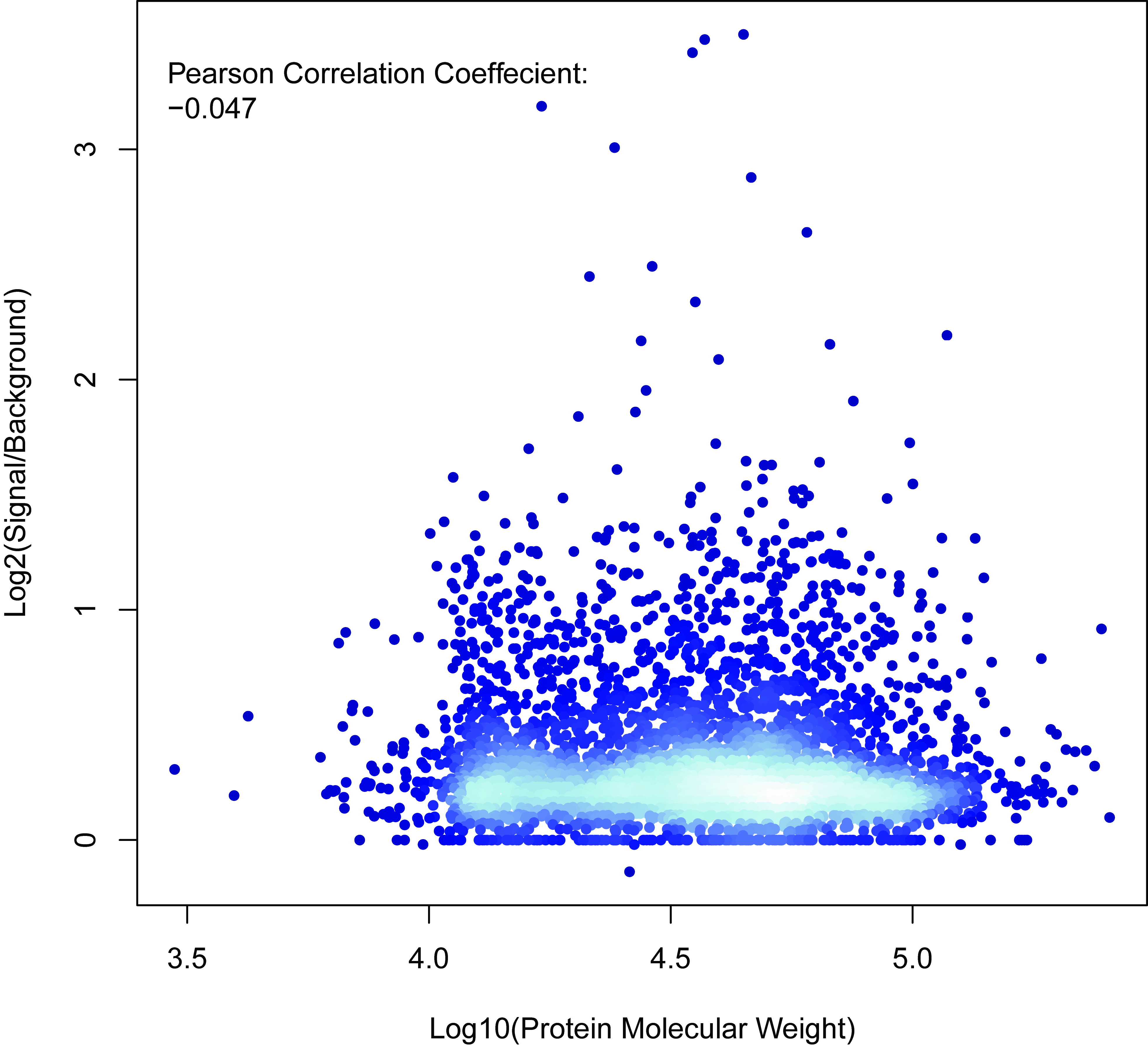

Supplement: Figure S1 — No bias based on protein size found in protein microarray data. Data for protein weight from [1]. (3.24 MB TIF) [file pone.0012671.s007.tif]

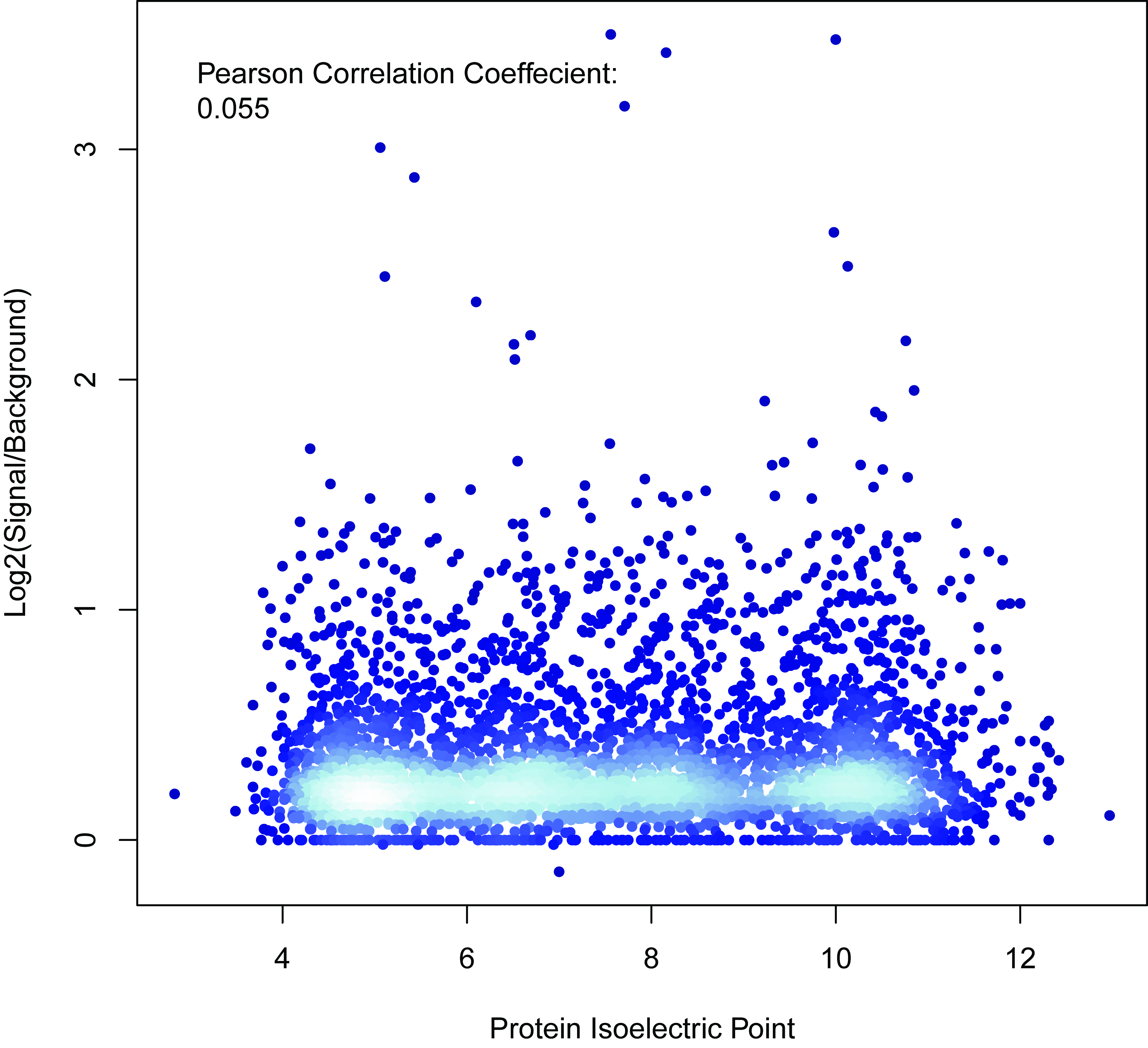

Supplement: Figure S2 — No bias based on protein charge found in protein microarray data. Data for protein weight from [2]. (3.41 MB TIF) [file pone.0012671.s008.tif]

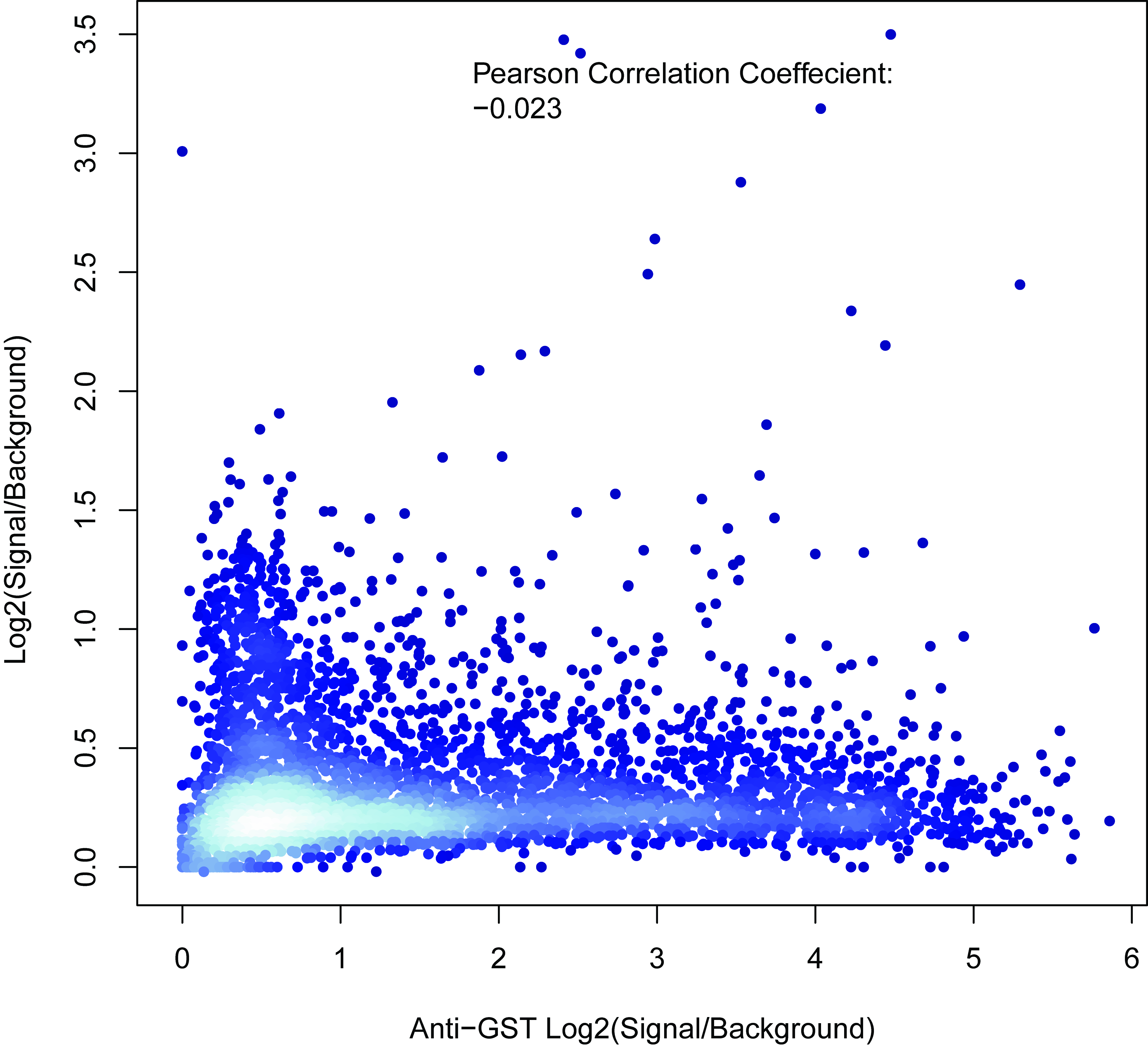

Supplement: Figure S3 — No bias based on amount of protein per spot found in protein microarray data. Amount of protein was estimated based on signal intensity of Hilyte Fluor 647-conjugated anti-GST antibody (1∶50) the protein arrays were probed with. (3.40 MB TIF) [file pone.0012671.s009.tif]

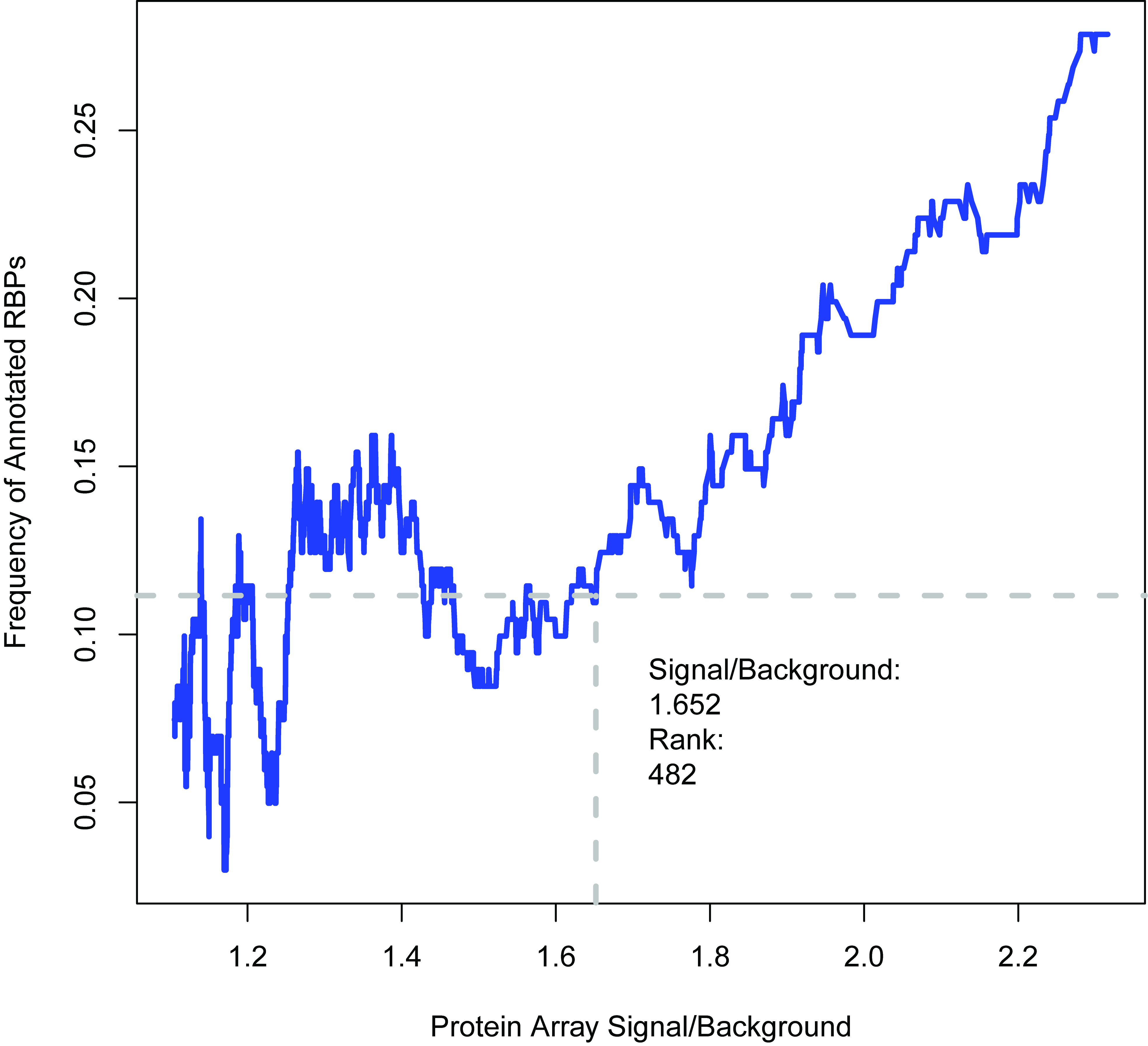

Supplement: Figure S4 — RBP Enrichment threshold for protein microarray data. A sliding window plot of 200 proteins is used. The gray line indicates the microarray signal-to-background value, at which the average frequency of annotated RBPs for the entire microarray is reached ( = 0.11), and the protein microarray data are not significantly enriching for RBPs. (1.79 MB TIF) [file pone.0012671.s010.tif]
